# Posterior laryngitis: a disease with different aetiologies affecting health-related quality of life: a prospective case–control study

**DOI:** 10.1186/1472-6815-13-11

**Published:** 2013-09-09

**Authors:** Hillevi Pendleton, Marianne Ahlner-Elmqvist, Rolf Olsson, Ola Thorsson, Oskar Hammar, Magnus Jannert, Bodil Ohlsson

**Affiliations:** 1 Department of Clinical Sciences, Division of Oto-Rhino-Laryngology, Skåne University Hospital, Malmö, Lund University, Lund, Sweden; 2 Department of Health Sciences, Lund University, Lund, Sweden; 3 Divison of Medical Radiology, Diagnostic Centre of Imaging and Functional Medicine, Skåne University Hospital, Malmö, Lund University, Lund, Sweden; 4 Division of Nuclear Medicine, Diagnsotic Centre of Imaging and Functional Medicine, Skåne University Hospital, Malmö, Lund University, Lund, Sweden; 5 Department of Clinical Sciences, Divison of Internal Medicine, Skåne University Hospital, Malmö, Lund University, Lund, Sweden; 6 Department of Oto-Rhino-Laryngology, Lasaretts gatan 21, Skåne University Hospital, Lund, SE-22185, Sweden

**Keywords:** Posterior laryngitis, Laryngo-pharyngeal reflux, Motilin, Functional gastrointestinal disorder, Health-related quality of life

## Abstract

**Background:**

Laryngo-pharyngeal reflux (LPR) is assumed to be the most common cause of posterior laryngitis (PL). Since LPR is found in healthy subjects, and PL patients are not improved by acid-reducing therapy, other aetiologies to PL must be considered. The aims of this study in PL were to investigate the prevalence of acid reflux in the proximal oesophagus and functional gastrointestinal symptoms, to analyse motilin levels in plasma, and to assess health-related quality of life (HRQOL) before and after treatment.

**Methods:**

Forty-six patients (26 women), with verified PL, median age 55 (IQR 41–68) years, were referred to oesophago-gastro-duodenoscopy and 24-h pH monitoring. Plasma motilin was analysed. The 36-item Short-Form questionnaire was completed at inclusion and at follow-up after 43±14 months, when also the Visual Analogue Scale for Irritable Bowel Syndrome was completed. Values were compared to controls. Treatment and relief of symptoms were noted from medical records.

**Results:**

Thirty-four percent had proximal acid reflux and 40% showed signs of distal reflux. Ninety-four percent received acid-reducing treatment, with total relief of symptoms in 17%. Patients with reflux symptoms had lower plasma motilin levels compared to patients without reflux symptoms (p = 0.021). The HRQOL was impaired at inclusion, but improved over time. Patients, especially men, had more functional gastrointestinal symptoms than controls.

**Conclusions:**

This study indicates that a minority of patients with PL has LPR and is cured by acid-reducing therapy. Disturbed plasma motilin levels and presence of functional gastrointestinal symptoms are found in PL. The impaired HRQOL improves over time.

## Background

Posterior laryngitis (PL) is defined as an inflammation involving the most posterior part of the glottic region, and sometimes involving the oesophageal inlet, in conjunction with symptoms such as chronic cough, hoarseness, a sensation of having a lump in the throat (globus), excessive throat clearing, excessive phlegm, voice fatigue, throat pain and dysphagia [1-4]. The pathogenesis of inflammation is multifactorial and smoking, alcohol abuse, viral or bacterial infections, allergy, chronic sinusitis, voice abuse, and laryngo-pharyngeal reflux (LPR) can be the underlying causes.

LPR is defined as a back flow of gastric contents into the laryngo-pharynx and can be established by objective measurements [5]. A previous study indicate that 4%−10% of the patients who visit a Department of Oto-Rhino-Laryngology have complaints related to LPR [2]. One study showed that as many as 50% of patients affected by laryngeal and voice disorders have a pH-documented reflux [1]. Although the exact mechanism is unknown, reflux is associated with oesophageal dysmotility in 50%–60% of cases and reduced pressure of the lower oesophageal sphincter (LOS) in the majority of cases [6-8]. The influences on the function of the LOS are numerous. Motilin is an intestinal hormone, which regulates the migrating motor complex (MMC) of the ventricle and affects the pressure of the LOS. The hormone is found in enterochromaffin cells in the duodenum and jejunum, and ingestion of fat and gastric acid stimulates secretion of motilin into the bloodstream [9,10]. Patients with reflux have been shown to have altered motilin levels [11].

Patients with PL are at risk of being over-diagnosed as having extra-oesophageal acid reflux, as LPR is the most often assumed aetiology of PL [12,13]. Consequently, an inappropriate use of proton pump inhibitors (PPIs) are prescribed even though the symptoms and the findings are not related to acid reflux [14]. It has recently been shown that patients with PL, despite treatment with sufficient doses of PPI over many years, still suffer from their initial symptoms and complaints, with impaired health-related quality of life (HRQOL) as a result [15].

Apart from acid reflux, functional heartburn, functional dyspepsia and functional oesophageal disease may lead to oesophageal complaints [16]. An overlap and comorbidity between different functional disorders often exists. This also applies to functional gastrointestinal disorders (FGID), one of the most common being irritable bowel syndrome (IBS) [17]. The reason to functional disturbances is unclear, but may depend on altered central processing of visceral afferent information [18], where patients with FGID are thought to have a heightened perception of normal visceral stimuli, called visceral hypersensitivity [19-21]. The same functional aetiology has also been suggested in the case of PL [13,15].

### Aim

The primary aim of the present study of PL was to investigate how many of the patients who had acid reflux in the proximal part of the oesophagus. Secondary aims were to examine motilin levels in plasma; to determine whether PL was associated with symptoms of FGID; and to register the HRQOL before and after treatment.

## Methods

This study was performed according to the Helsinki declaration, and was approved by the Regional Ethics Review Board at Lund University. Informed, written consent was obtained from the participants.

### Subjects

All consecutive patients, >18 years of age, from the Department of Oto-Rhino-Laryngology, Skåne University Hospital, Malmö, were invited during the period June 2007 to May 2011 to participate in the study when the diagnosis PL was made by fibre optic laryngoscopy of an examiner not blinded to the patients symptoms. The diagnosis criteria for PL [1-4], and thereby the criteria for inclusion in the study, were the thickening and/or oedema of the posterior part of the glottic region *in combination* with one or several of the following symptoms: chronic cough, hoarseness, a sensation of having a lump in the throat (globus), excessive throat clearing, excessive phlegm, voice fatigue, throat pain and dysphagia. Patients with signs of PL but no symptoms were not enrolled in the study. Additional exclusion criteria were inflammatory bowel disease (IBD), coeliac disease, pregnancy, serious illness such as severe heart, lung, liver or kidney diseases and mental illness. Patients with Hepatitis B, C and HIV/AIDS were not included.

### Study design

At the time of inclusion, age, symptoms and findings typical of the diagnosis PL, duration of symptoms, previous treatment for acid-related disease and symptoms, and tobacco and alcohol habits were registered. The patients were asked to fill in the 36-item Short-Form questionnaire (SF-36) to evaluate HRQOL. All patients were referred to a single-probe, 24-h pH monitoring in the proximal part of the oesophagus and an oesophago-gastro-duodenoscopy (OGD). An instant test for *Helicobacter Pylori* was made at OGD when clinically indicated. Blood samples were collected for the analysis of motilin.

At follow-up 43 ± 14 months later, a letter including the questionnaire SF-36 and the Visual Analogue Scale for Irritable Bowel Syndrome (VAS-IBS) was sent to the patients. Medical records were scrutinized and information such as choice of treatment (kind of drug, dosage and duration of treatment) and the extent of relief of symptoms were noted.

### Ambulatory 24-h pH monitoring

All participants were instructed to cease their PPI therapy seven days before, and other acid inhibitors 16 h before the monitoring. They were asked to avoid acid beverages, e.g. fruit juice, during the 24-h duration of the pH monitoring and to fast 4 h before the catheter was introduced. The positioning of the catheter was performed in the Diagnostic Centre of Imaging and Functional Medicine, with the aid of fluoroscopy (Philips Multidiagnost Eleva, CA, USA). The catheter was introduced through the nose and under fluoroscopic control positioned in the proximal part of the oesophagus, 5 cm below the upper oesophageal sphincter.

Ambulatory pH monitoring for proximal reflux was performed for 24 h. Oesophageal pH monitoring was performed using an antimony pH electrode with an internal reference electrode (Versaflex, Sierra Scientific Instruments, Los Angeles, Ca, USA). Before each study, the pH-probe was calibrated in buffer solutions of pH 7 and 1. An episode of acid reflux was defined as a decrease in oesophageal pH to below 4 for more than 10 s. Previously established upper limits of normal acid exposure in clinical studies, with pH < 4 for 1% of total time, were used in the analysis of the data [22,23]. The data were stored on a portable digital recorder (Digitrapper pH400, Synectics Medical, Stockholm, Sweden) and were analysed with commercially available software (Polygram NET, SynMed Medical, Stockholm, Sweden).

### Measurements of motilin

After fasting for at least 9 h, blood samples were taken at the time of the OGD. All blood samples consisted of 8.0 ml whole blood drawn into heparinised tubes. The plasma was separated and frozen at −20°C within 1 h of collection. Plasma motilin was measured as previously described by a radioimmunoassay (RIA) using a rabbit antiserum (R-8423), raised against highly purified, porcine motilin. The antiserum is directed to the NH2-terminal of motilin (amino acids 1–9). The coefficient of variation, intra-assay CV, was < 8% for controls at 140 pmol/l [24].

### Questionnaires

#### The 36-item short-form questionnaire

SF-36 is an extensively used HRQOL instrument, which provides reproducible, reliable data on large populations, and has been shown to be useful as a global health monitor in clinical practice [25]. It is available in Swedish [26], and Swedish reference data are available for many different conditions. The SF-36 questionnaire is divided into eight subscales of general health, arranged according to the degree to which they measure physical vs. mental health. These subscales are physical functioning (PF), role functioning-physical (RP), bodily pain (BP), general health (GH), vitality (VT), social functioning (SF), role functioning-emotional (RE), and mental health (MH). Two additional dimensions can be calculated, physical (PCS) and emotional health (MCS), based on the weighting of the importance of the other eight subscales. The raw data were recoded at the time of analysis; the maximum score is 100, the higher score the better the HRQOL.

#### The visual analogue scale for irritable bowel disease

The VAS-IBS questionnaire is designed to measure the symptoms, the response to the treatment, and well-being in patients suffering from IBS. It was previously developed and validated for patients with gastrointestinal symptoms without organic causes [27]. Patients estimated seven different aspects of their gastrointestinal condition on a visual analogue scale (VAS) from 0–100 mm, where 0 represents very severe problems and 100 represents absence of problems. The aspects are abdominal pain, diarrhoea, constipation, bloating and flatulence, vomiting and nausea, perception of psychological well-being and the intestinal symptoms' influence on daily life. It also contains two additional questions concerning the patient's sense of urgency to defecate and the feeling of incomplete evacuation after defecation, which can be answered by “yes” or “no”.

### Statistical analyses

The data were analysed using the statistical software package SPSS for Windows^©^ (Release 20.0; IBM). Distribution among the study population was tested by the Kolmogorov-Smirnov test and differed significantly from a normal distribution (p < 0.001) regarding age, VAS-IBS, and motilin levels. Results are given as means and the standard error of the mean (SEM) or median and interquartile range (IQR). Differences between groups were calculated, when appropriate by the Mann–Whitney U-test. Fisher’s exact test was used for categorical variables and Spearman rank correlation test was used for correlations. The one-sample t-test was used to compare data from the SF-36 questionnaire with the Swedish reference values. Values were compared with the norm values of the general, and the Swedish female and male population, corrected for age [26]. As the patient group was older than the controls for VAS-IBS, the VAS variables were age-standardized using a linear regression model into which age was added as a covariate (independent). The dependent variables abdominal pain, diarrhoea, constipation, bloating and flatulence, vomiting, nausea, perception of psychological well-being, and the intestinal symptoms' influence on daily life were expressed as z-scores. As symptoms differ between gender [28], the results for women and men were calculated separately. Logistic regression analysis, adjusted for age (divided into 5-year intervals) and gender, was used to calculate odds ratios with 95% confidence intervals (OR with 95% CI) for the prevalence of the patient's sense of urgency to defecate and whether the bowel was totally emptied after defecation. The level of statistical significance was set to p ≤ 0.050.

## Results

### At inclusion

#### Patient characteristics

**Figure 1:**
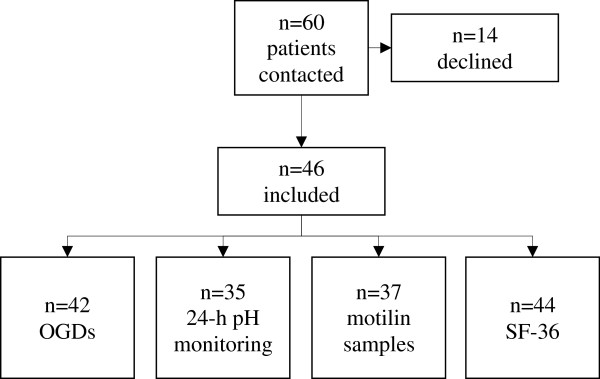
Flow-chart illustrating the selection process and the number of procedures among the included patients.

Forty-six out of 60 invited patients with verified PL (26 women), median age 55 (IQR 41–68) years, accepted to participate and were included in the study. Fourteen patients did not want to participate, the majority declining without giving a reason. Thirty-five patients tolerated the 24-h pH monitoring, 37 patients agreed to give blood samples for analysis of motilin, 42 patients were examined by OGD, and 44 patients filled in the SF-36 questionnaire (Figure 1). The mean duration of the disease was 13.5 (range 0–180) months. The most common symptoms registered were globus (61%), excessive phlegm (48%), hoarseness (37%), voice fatigue (26%), and heartburn (26%) (Table 1). At fibre laryngoscopy, signs of PL such as inter-arytenoid pachydermia (76%), arytenoid oedema (56%), post-cricoid oedema (22%), and erythema of the vocal folds (11%) were found. Seventeen (11%) of the patients were smokers and 14 (9%) had been treated for dyspepsia, gastro-oesophageal reflux disease (GORD) or ulcer before their visit to the ear, nose and throat specialist. Five patients had a previously found hiatal hernia. Information about drinking habits was lacking in the majority of the cases.

**Table 1:** The prevalence of the various symptoms in the patients with posterior laryngitis (n=46)

**Symptoms**^**a**^	**Number and percentage of patients**
Globus	28 (61)
Excessive phlegm	22 (48)
Hoarseness	17 (37)
Heartburn	12 (26)
Voice fatigue	12 (26)
Acid regurgitation/reflux	11 (24)
Coughing	11 (24)
Excessive throat clearing	11 (24)
Dysphagia	9 (20)
Breathing difficulties	1 (2)

^a^More than one symptom for each patient was registered.

#### Ambulatory 24-h pH monitoring and OGD

Thirty-four percent, 12 of the 35 patients examined (6 women), median age 55 (IQR 30–70) years, had a pathological reflux to the proximal oesophagus. Voice problems were associated with a pathological 24-h pH monitoring result (p = 0.050).

No ulcerations or tumours were found at OGD, but seven suffered from oesophagitis, 10 from Barrett's oesophagus, 14 from hiatal hernia and 14 had normal OGDs. Of the 12 patients complaining of voice problems, 11 were examined by OGD. The results of this procedure showed that these patients suffered from one or several of the following findings: five patients had a hiatal hernia, three suffered from Barrett’s metaplasia, two had oesophagitis and one patient was positive for *Helicobacter Pylori*. Two patients had normal OGDs.

#### Measurements of motilin

Thirty-seven patients (20 women), median age 55 (IQR 40–67) years, gave blood samples for the analysis of motilin. Twenty-one healthy volunteers (14 women), median age 43 (IQR 36–53) years, served as controls. The motilin levels showed no correlations with age (r_s_ = 0.180, p = 0.287). There were no differences in plasma levels of motilin between patients and controls (p = 0.517), but the motilin levels differed between patients reporting typical reflux symptoms, i.e. heartburn and/or regurgitations (median 61 (IQR 52–68) pmol/l), compared to those without reflux symptoms (median 71 (IQR 64–85) pmol/l) (p = 0.021). There were no differences in plasma levels of motilin between patients with a positive or negative 24-h pH monitoring result (p = 0.057) or between patients with a pathological or normal OGD (p = 0.441).

#### SF-36

**Figure 2:**
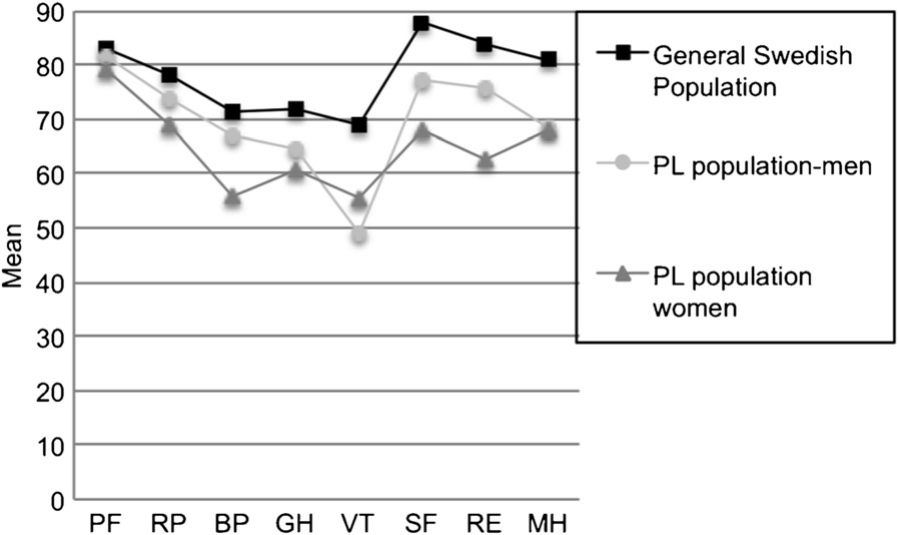
**Analysis of the SF-36 questionnaire at inclusion in the population of men and women with posterior laryngitis (PL) and the general Swedish population.** Gender- and age-matched values are presented as mean values. PF = physical functioning, RP = role-physical, BP = bodily pain, GH = general health, VT = vitality, SF = social functioning, RE = role-emotional, ME = mental health. One-sample t-test. P ≤ 0.050 was considered statistically significant.

The women had significantly lower scores for all subscales, except PF that did not differ from the Swedish female population (p = 0.264) or the general Swedish population (p = 0.116) (Figure 2). In men, no differences in scores were found compared to the general Swedish population (Figure 2). The score for MH was significantly lower in the PL group compared to the Swedish male population (p = 0.050). The total PL population (n = 44) had significantly lower scores for all subscales except PF and RP, compared to the general Swedish population (data not shown).

#### Treatment results

Forty-three patients (94%) received acid-reducing treatment. The most common treatment prescribed was PPIs alone or in combination with alginic acid (Table 2). The preferred choice of dose and duration of treatment was 20 mg PPI twice daily for 2–3 months. About one-fifth of the patients were treated for 8 weeks or less and 16% were treated for 4 weeks or less. At the clinical follow-up, more than half of the patients were better, but only 17% were asymptomatic (Table 2). An appointment with the physician, with a renewed fibre optic examination of the larynx was the most common mode of follow-up (52%), whereas 44% of the patients were followed by a telephone consultation. Two patients had no follow-up.

**Table 2:** Medical treatment and results of treatment (n = 46)

	**Patients n (%)**
**Medical treatment**^**a**^	
Proton pump inhibitor	41 (89)
Alginic acid	13 (28)
Histamine receptor blocker	2 (4)
Aluminum dihydroxide	0 (0)
Missing information	3 (7)
**Relief of symptoms**	
Complete lack of symptoms	8 (17)
Improved	26 (57)
No change	9 (20)
Worse	1 (2)
Missing information	2 (4)

^a^Some patients were treated with more than one drug.

### Follow-up

At study follow-up, 40 patients (22 women), median age 61 (IQR 44–72) years, completed both SF-36 and VAS-IBS.

#### SF-36

**Figure 3:**
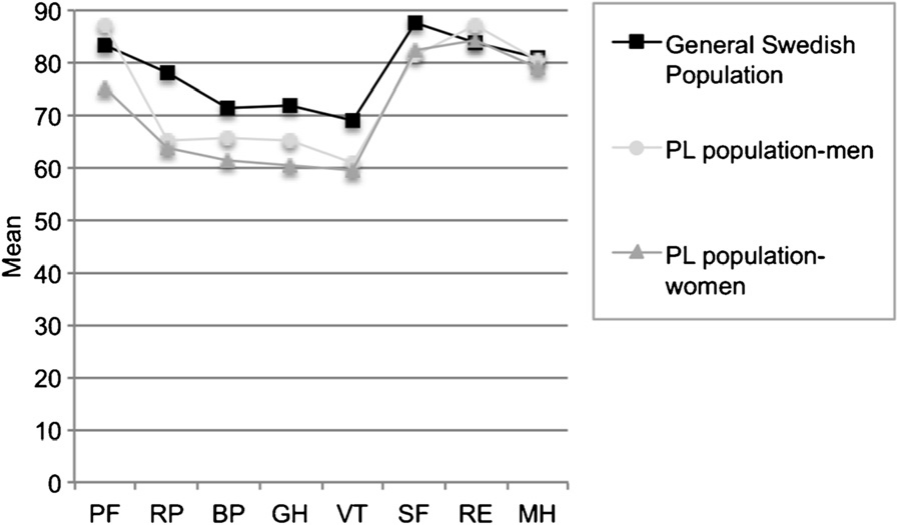
**Analysis of the SF-36 questionnaire at follow-up in the population of men and women with posterior laryngitis (PL) and the general Swedish population.** Gender- and age-matched values are presented as mean values. PF = physical functioning, RP = role-physical, BP = bodily pain, GH = general health, VT = vitality, SF = social functioning, RE = role-emotional, ME = mental health. One-sample t-test. P ≤ 0.050 was considered statistically significant.

The women had a significantly lower score for the subscale GH compared to the general Swedish population (p = 0.046) (Figure 3), but no significant difference was noted compared to the Swedish female population (data not shown). In men, no differences in scores were found compared to the general Swedish population (Figure 3) or to the Swedish male population (data not shown). The total PL population had significantly lower scores in the subscales RP (p = 0.047), GH (p = 0.016), and VT (p = 0.028) compared to the general Swedish population.

#### Visual analogue scale for irritable bowel syndrome

**Figure 4:**
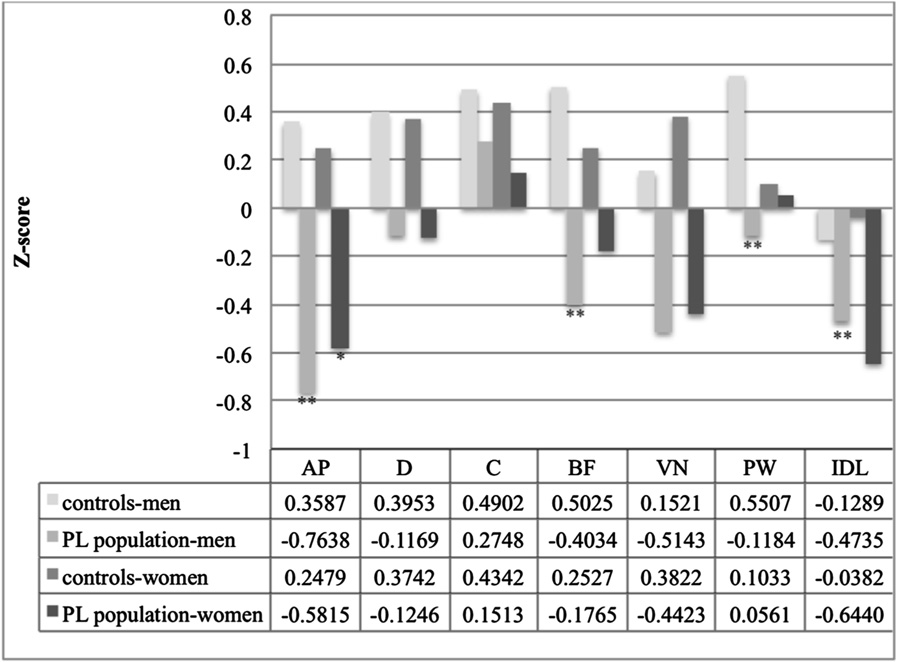
**Analysis of the VAS-IBS questionnaire, expressed as median z-scores, for the PL group and the controls at follow-up.** AP = abdominal pain, D = diarrhoea, C = constipation, BF = bloating and flatulence, VN = vomiting and nausea, PW = perception of mental well-being, IDL = intestinal symptoms effect on daily life. Mann–Whitney U-test. P ≤ 0.050 was considered statistically significant. * = Significant difference to controls-women, ** = Significant difference to controls-men.

Eighty-three volunteers (65 women), median age 39 (IQR 35–44) years, not suffering from PL or severe organic disease, served as controls for the VAS-IBS questionnaire. Women in the PL group registered significantly lower scores than the controls concerning abdominal pain (AP) (p = 0.001). The men in the PL group rated their symptoms as more severe than the controls, with a significant difference concerning abdominal pain (AP) (p<0.001), bloating and flatulence (BF) (p = 0.042), perception of psychological well-being (PW) (p = 0.024), and their intestinal symptoms' influence on daily life (IDL) (p = 0.020) (Figure 4). A positive correlation was found between emotional health (MCS) and perception of psychological well-being (WB) (r_s_ = 0.542, p = 0.001) and intestinal symptoms' influence on daily life (IDL) (r_s_ = 0.423, p = 0.009). There was no correlation between physical health (PCS) and the VAS-IBS (data not shown).

There was no significant difference between the PL group and the controls concerning the patient's sense of urgency to defecate (OR = 1.497, 95% CI = 0.233−9.605, p = 0.670) or the feeling of incomplete evacuation after defecation (OR = 1.494, 95% CI = 0.340−6.569, p = 0.595).

## Discussion

The majority of the patients with PL in the present study had been prescribed acid-reducing treatment. However, only a minority of the patients were asymptomatic at clinical follow-up. Although the patients were perceived as having LPR-induced inflammation, only 34% of the patients with typical signs and symptoms of PL had a proximal acid reflux and associated voice problems. At OGD, approximately 40% displayed signs of distal reflux. There was no difference in plasma levels of motilin between patients and controls, but patients with reflux symptoms had lower motilin levels compared to those without these symptoms. The total PL population's HRQOL was improved during the study, and the women's HRQOL improved more than the men’s. The PL group, especially the men, experienced functional symptoms from the gastrointestinal tract to a greater extent than controls.

The terminology concerning PL and LPR is unclear and the terms are used synonymously. Patients included in studies are defined to have LPR when they actually have signs and symptoms of PL and vice versa [29,30]. Laryngo-pharyngeal reflux has been observed in healthy subjects without any association to typical symptoms of PL [31-34]. Further, Ylitalo et al. [35] could not prove a significant difference in the occurrence of any specific pharyngeal or laryngeal symptom or finding between the patients with and without extra-oesophageal reflux in patients with chronic heartburn. Although LPR has been shown to be more prevalent in PL patients than in controls, LPR does not render specific laryngeal symptoms [32]. This indicates an incomplete concordance between symptoms, objective findings and reflux in PL, and that the diagnosis cannot be based on symptoms alone [5,32]. Nevertheless, it is assumed that most patients suffering from PL have reflux of gastric content into the pharynx and larynx, LPR.

In the current study, all patients included displayed typical findings and symptoms of PL, but only 34% of the patients had a pathological 24-h pH value. Still, the majority of our patients received anti-reflux treatment with PPIs. Only 17% of the patients reported disappearance of symptoms at the clinical follow-up. Almost half of the patients were followed by a consultation by telephone. As a previous study has displayed that symptoms of PL treated with PPI resolve before laryngeal lesions heal, we assumed that a telephone consultation after 2–3 months of treatment is a sufficient mode of follow-up in the majority of cases [36]. At the telephone consultation, if the treatment has failed to improve the patient's symptoms, a new evaluation should be performed and additional aetiologies of PL, e.g. allergy, voice abuse, viral or bacterial infection, insufficient doses of PPI or functional disease should be considered.

Patients suffering from functional oesophageal disease are known to display typical reflux symptoms (heartburn/regurgitations) in spite of a normal acid exposure time and a normal oesophageal mucosa [19]. This is considered to be due to visceral hypersensitivity, i.e. an enhanced perception of normal physiological signals arising from the oesophagus. Different functional disorders often overlap, and patients may suffer from more than one such disorder [37]. These patients have a poorer response to acid suppressive therapy [3,19], which also applies to acid-suppressing therapy to patients with PL [15]. In accordance with patients affected by functional oesophageal disease, patients diagnosed with PL might suffer from functional pharyngeal-laryngeal disease. Patients with FGID are known to have a comorbidity of affective disturbances, e.g. depression and anxiety [38], which also has been noted in patients with PL [30]. The likelihood of a functional component is further strengthened by the expression of serum antibodies against gonadotropin-releasing hormone in patients both with PL and IBS [39,40].

The men had lower scores concerning abdominal pain, bloating and flatulence compared to the controls, whereas women only had lower scores for abdominal pain, indicating that patients with PL suffer from more gastrointestinal symptoms than the controls. Women have more gastrointestinal symptoms than men in the general population [28,41]. On the contrary, Sadik et al. [42] found in a group of patients with severe unexplained gastrointestinal symptoms, that gastrointestinal transit abnormalities were more common in men. The VAS-IBS results in our study are in agreement with these results [42], and suggest that the prevalence of gastrointestinal disorders may not differ that much between genders, when efforts are made to objectively assess the complaints.

The present study could not prove a difference in plasma levels of motilin between patients and controls, but patients with reflux symptoms had lower levels of motilin compared to those without these symptoms. Altered plasma levels of motilin have been observed in patients with GORD, and motilin has in vitro been shown to elicit contractions of the LOS [10,11]. Patients with GORD, defective LOS pressure, and oesophageal dysmotility have been found to have lower plasma motilin levels than patients with normal peristalsis, and the motilin levels, LOS pressure and oesophageal dysmotility were normalised after anti-reflux surgery [43]. We have recently shown in a group of diabetic patients that plasma motilin concentrations vary with abnormalities in oesophageal motility [44], and the results of the present study raise the hypothesis that motilin might be of importance for the LOS function and oesophageal motility in LPR-induced PL. Basal and peak plasma levels of motilin, 24-h combined multi-channel intra-luminal impedance and pH testing (MII-pH) or 24-h pH monitoring with dual probes, would be of interest to further determine the role of motilin in patients with PL.

Siupsinskiene et al. [30] reported a significant deterioration in HRQOL in PL patients. Carrau et al. [29] also demonstrated, by using the SF-36 questionnaire, that patients with symptoms of LPR have a significantly inferior HRQOL than the general U.S. population.

In the present study, HRQOL was improved at the follow-up after 43 months, compared to the initial assessment. The absence of correlation between physical health (PCS) and the physical items of the VAS-IBS confirms that the impaired HRQOL in our patients with PL are not due to FGID, but due to PL. The lower HRQOL of women at the start of the study might reflect the propensity of women to complain about their symptoms [28,41,45]. Subsequent normalisation of their HRQOL at follow-up might be due to adequate treatment, receiving a diagnosis and information about the nature of their disease, or being taken care of. Bengtsson et al. [46] have shown that being taken care of, and information/education, improves women's quality of life when suffering from functional diseases.

One limitation of this study is the small group of patients studied. Also, we have not excluded the possibility that our patients might suffer from weakly acidic, gaseous or non-acidic reflux, as we have registered their reflux only by pH monitoring with a single probe, and not with combined multi-channel intra-luminal impedance and pH testing (MII-pH) or 24-h pH double probe monitoring. Our controls for motilin and VAS-IBS were entirely or partly recruited from hospital staff, who may be healthier than the average individual, and our control and study groups might have gained from being better matched. We have tried to compensate for this shortcoming by statistical adjustments of the data.

## Conclusion

This study indicates that patients with typical findings and symptoms of PL to a lesser extent have LPR, and that PL, in a subgroup of patients, may be due to functional disease. Voice problems were associated with pathological 24-h pH values. The plasma level of motilin is affected among PL patients with typical reflux symptoms, suggesting that this hormone is important in the pathogenesis of LPR. The HRQOL of the patients, especially the women, was low initially, but improved over time. It is highly probable that there are subgroups within PL, where some patients may have LPR, some display dysmotility of the oesophagus, and some patients have a functional disease.

## Abbreviations

FGID: Functional gastrointestinal disorders; GORD: Gastro-oesophageal reflux disease; HRQOL: Health-related quality of life; LOS: Lower oesophageal sphincter; LPR: Laryngo-pharyngeal reflux; OGD: Oesophago-gastro-duodenoscopy; PL: Posterior laryngitis; PPI: Proton pump inhibitor; SF-36: 36-item Short-Form questionnaire; VAS-IBS: Visual analogue scale for irritable bowel syndrome.

## Competing interests

The authors declare that they have no competing interests.

## Authors’ contributions

HP, BO, MJ and MAE together designed the study. HP has substantial contribution to study conception, acquisition of data and for mainly drafting the manuscript. HP and BO are mainly responsible for analysis and interpretation of data. RO collected the data from Medical Radiology, Diagnostic Centre of Imaging and Functional Medicine. OT collected the data from Nuclear Medicine, Diagnostic Centre of Imaging and Functional Medicine. OH collected data concerning the controls for VAS-IBS from the Department of Internal Medicine. All authors contributed to the manuscript with constructive criticism, and read and approved the final manuscript.

## Pre-publication history

The pre-publication history for this paper can be accessed here:

http://www.biomedcentral.com/1472-6815/13/11/prepub
